# Impact of Kosher Slaughter Methods of Heifers and Young Bulls on Physical and Chemical Properties of Their Meat

**DOI:** 10.3390/foods11040622

**Published:** 2022-02-21

**Authors:** Jagoda Żurek, Mariusz Rudy, Paulina Duma-Kocan, Renata Stanisławczyk, Marian Gil

**Affiliations:** Department of Agricultural Processing and Commodity Science, Institute of Food and Nutrition Technology, College of Natural Sciences, University of Rzeszow, Zelwerowicza 4, 35-601 Rzeszów, Poland; jzurek@ur.edu.pl (J.Ż.); pduma@ur.edu.pl (P.D.-K.); rstanislawczyk@ur.edu.pl (R.S.); mgil@ur.edu.pl (M.G.)

**Keywords:** beef, ritual slaughter, young bulls, heifers, meat quality

## Abstract

This work aimed to comprehensively analyze the factors (slaughter method, gender, and muscle type) that determine the kosher status of beef and assess their influence on the selected quality characteristics of raw meat. The muscles were obtained from 40 carcasses of heifers and 40 carcasses of young bulls. In the first stage of the experiment, pH values were measured. The water, protein, fat, minerals, and collagen contents were determined. Then, the shear force, forced drip, and thermal drip were measured. The experimental results indicated that all the investigated parameters have an impact on the final quality of beef. Statistically significantly lower pH_1_ values were noticed in the *longissimus thoracic* muscle of young bulls obtained through kosher slaughter methods. However, 24 and 48 h after slaughter, higher pH values were observed in the meat of young bulls obtained by the kosher slaughter method, where the meat samples were subjected to kosher treatment. The koshering process (salting and washing) resulted in a significant reduction in both forced and thermal drip values of the meat sample, but this decrease was not affected by gender.

## 1. Introduction

Stress is the most frequently identified factor in the handling of animals prior to slaughter, which negatively affects the quality of meat. Pre-slaughter stress and energy inputs deplete muscle glycogen reserves and, as a result, cause insufficient post-mortem production of hydrogen ions. The end products of ATP hydrolysis and post-mortem glycolysis, hydrogen ions and lactate, accumulate in the muscle due to the lack of an effective elimination mechanism. This accumulation of hydrogen ions acidifies the muscles and consequently causes drop of pH [1]. Low acidity during maturation changes the color, taste, and tenderness of meat [2,3,4]. Significant pre-slaughter stress also affects the firmness and ability to retain water, but also reduces the tenderness of meat [5].

The weather conditions in the pre-slaughter period may increase additional stress for the animals. Seasonal temperature changes can affect muscle glycogen levels after slaughter and the final pH. The increase in glycolysis results from excessive excitement, hunger, and stress caused by the ambient temperature, leading to high post-mortem pH values [4,6,7]. The conditions for keeping cattle in livestock warehouses also have a great influence on meat quality [8].

The concentration of glycogen in the muscles at the time of slaughter is one of the most important factors determining the quality of beef. Insufficient glycogen reserves during slaughter lead to pH values higher than 5.5 [3]. Meat characterized by high pH values is dark and more susceptible to bacterial spoilage, and it is less durable [2]. The problem of reduced meat quality caused by pre-slaughter procedure occurs more often in the meat of young bulls than heifers [8].

The scientific literature provides detailed information regarding the specifications of various types of ritual slaughter methods that are commonly practiced for the slaughtering of cattle and other animal species [9,10]. Kosher slaughter is performed by a qualified butcher (known as a shochet) and involves continuous cutting of the esophagus and blood vessels using a special sharp chalef knife, with the length of the straight blade being at least twice the diameter of the animal’s neck [11,12,13]. The shochet slaughters the fully conscious animal and examines the cut on the animal’s neck after each slaughter to make sure that the cut is carried out “perfectly” [13]. If the blade has a nick or is otherwise damaged, the animal is considered to be “tref” or not kosher, and the meat obtained from this process is sold in the regular market [14]. Additionally, the shochet performs a post-mortem examination of the carcasses to detect any changes, especially in the chest, lungs, and liver. If disease symptoms are observed, the meat of such an animal may not be considered suitable for consumption [9,15,16]. In addition, inappropriate cutting may produce non-kosher meat, which is not suitable for consumption by consumers who specifically eat kosher meat [16]. After slaughtering is completed, the meat is further processed by efficiently removing certain veins and arteries, forbidden fat, and blood. In the United States and most of the Western countries, only the front quarters of beef are used [13]. Koshering is the final step in the process of making the meat suitable for consumption [17]. The term “koshering meat” refers to the meat that is obtained from the animals that are subjected to certain rituals before slaughtering and is followed by the rabbi’s inspection of the carcass to detect the presence of any irregularities. If the carcass passes the inspection, then it is classified as “kosher.” The meat from the certified carcasses is soaked in water for half an hour, salted with coarse salt for 1 h, and finally rinsed with water three times [18,19].

A significant reduction in pH values has been observed for meat slaughtered by the kosher method when compared with non-kosher meat samples [20]. In addition, cold water soaking of the raw meat (30 min) and subsequently salting its surface with coarse salt (approximately 1 h) [19] have been shown to help in the removal of myoglobin and other sarcoplasmic proteins during the koshering process [21]. Partially removing the myoglobin affects the color, taste, and overall quality of the finished product; however, from a health perspective, it is its influence on the oxidation processes that is the most important effect [22]. In addition, a reduction in the concentration of heme proteins influences the final product color. It has been shown that kosher meat has a low color intensity [20]. Moreover, an important factor that contributes to the enhanced kosher meat nutritional quality in comparison with the meat from standard slaughter methods is its high salt content [23]. Previous have studies investigated the influence of breed [24], gender [25], age [26], muscle type [27], and various environmental and genetic factors [28] on the final quality of meat. Furthermore, the quality of beef is determined by the procedures that are followed at all stages of meat production, starting from the appropriate selection of the breed, safety measures adopted during rearing, transport to the slaughterhouse, humane slaughter, cooling of the carcasses, and finally maintaining optimal conditions for tenderization and distribution of the end product [8].

However, few studies in the literature exclusively describe the impact of factors on the quality characteristics of beef obtained by kosher slaughter method. Owing to the fact that meat is a product that shows high variability in its characteristics, which can be attributed to the interactions between numerous genetic and environmental factors, the previously conducted studies in different environments and for variable periods of time do not provide a clear solution for the problem posed at this time [29]. The present study may show new outcomes or confirm the results obtained by previous studies.

Taking into account the above-mentioned information, research was carried out to comprehensively analyze the factors (slaughter method, gender, and muscle type) that influence the kosher status and nutritional quality of beef and assess their impact on the selected quality characteristics of raw meat. This knowledge will enable us to explore novel and effective methods to obtain a finished product with selected characteristics and nutritional quality.

## 2. Materials and Methods

### 2.1. Raw Material

The study sample consisted of two types of muscles: the *longissimus thoracic* muscle (*musculus longissimus thoracis*, MLT) and *supraspinatus* muscle (*musculus supraspinatus*, MS). The muscles were obtained from 40 carcasses of heifers (the average live weight of 20 heifers selected for traditional slaughtering was 520 ± 58 kg, and the average live weight of 20 heifers selected for kosher slaughtering was 531 ± 60 kg) and 40 carcasses of young bulls (the average live weight of 20 young bulls selected for traditional slaughtering was 591 ± 52 kg, and the average live weight of 20 young bulls selected for kosher slaughtering was 571 ± 59 kg). The age of the cattle was 20–24 months, and the experimental animals were obtained by crossing the Polish Holstein–Friesian breed cows with the Limousine breed bulls. All the animals came from one breeder, were bred and raised on a single farm, and were enclosed in a semi-intensive system. In the summer, the basic animal feed comprised green matter of grasses and maize silage, and in the winter, it was predominantly maize silage. The animals were given additional supplements in the form of meadow hay and ground grain. Detailed information about the animals was obtained from the purchase documents of the slaughterhouse. The cattle were transported to a meat processing plant in south-eastern Poland, and were kept in a livestock warehouse in single pens for 20 h. After weighing, the animals were slaughtered according to the meat industry protocol.

Two types of slaughter techniques were used to obtain the muscle samples:

1. Standard procedure, which involves stunning the animals mechanically using a pneumatic captive bolt pistol (40 heads);
2. Ritual procedure, in which slaughtering is performed in specially designed boxes (without stunning), and after 24 h of cooling down the carcasses, the muscle samples are subjected to kosher treatment, which involves the preliminary rinsing of quarters in water under specific conditions, salting, and rewashing three times (40 heads).

Kosher slaughter and standard slaughter were performed on different days. Beef obtained from carcasses of both genders, killed by standard slaughter protocol, were not subjected to koshering.

The animal remains obtained from both traditional and kosher slaughtering methods were subjected to electrical stimulation for 15 min under the following conditions: voltage, 21 V; current, 18 mA; and impulse duration comprising 3.5-s impulse, 1-s pause, 3.5-s impulse, 1-s pause, and 3.5-s impulse. The carcasses were evaluated according to the EUROP classification system. Based on the conformation of the carcass, 50% of the samples were graded as R (good) and the remaining 50% as O (fair) in both traditional and kosher slaughter, and in terms of fatness, all the samples (100%) were graded as Class 3 (average).

### 2.2. Analytical Methods

In the first stage of the experiment, pH values were measured in the selected beef half carcasses (left side) obtained from both traditional and ritual slaughter methods. Measurements were performed in the MLT and MS. The first measurement was taken on a warm half carcass about 1 h after slaughter. The subsequent measurements were obtained after 24 (after kosher treatment in the case of muscles obtained from carcasses of cattle from kosher slaughter) and 48 h of cooling at 0 °C–2 °C. After 72 h, the quarters were cut into different sections. During post-slaughter chilling, half carcasses obtained from ritual and standard slaughter methods were stored in separate cooling rooms built for this purpose. All experiments and measurements were carried out in rooms under a controlled temperature by maintaining the temperature in the range of 0 °C–2 °C. After 48 h of slaughtering, about 0.5 kg of sample was collected from individual muscles for analysis by laboratory tests (2 slaughter types × 2 sex groups × 2 muscle groups × 20 carcasses = 160 samples).

The pH of the meat was measured using a pH meter pH-K21 (NWK-Technology GmbH, Aichach, DE, Germany) equipped with a LoT406-M6-DXK-S7/25 electrode from Mettler Toledo GmbH, Greifensee, CH, Switzerland. The electrode was driven into the muscles up to a depth of 25 mm. The probe was calibrated against buffers with pH values of 6.88 and 4.00. The measurement was carried out with an accuracy of 0.01.

Water content was determined according to PN-ISO 1442:2000 standard [30].

Protein content was determined using the Kjeldahl method, and the calculated amount of nitrogen was converted into crude protein according to PN-A-04018 [31].

Fat content was determined using the Soxhlet method in accordance with PN-ISO 1444: 2000 [32], and the salt content was determined by the Mohr method according to PN-A-82112 [33]. The amount of collagen protein was determined based on the content of hydroxyproline (conversion factor 8) according to PN-ISO 3496: 2000 [34], using the ultraviolet–visible Spekol 2000 spectrophotometer (Analytik Jena AG, Jena, DE, Germany).

The mineral content was expressed as total ash and determined according to the guidelines mentioned in PN-ISO 936: 2000 [35] using the LECO TGA701 thermogravimetric analyzer (Leco, St. Joseph, MI, USA).

To further determine the effect of physicochemical characteristics, that is, thermal drip and forced drip, the meat samples were minced twice in a laboratory mincer (Zelmer, Rzeszów, PL, Poland) and filtered using sieves of 4-mm mesh size. The obtained meat mass was mixed thoroughly to homogenize the sample.

The size of thermal drip (meat samples were cooked in a water bath at water temperature 85 °C for 10 min) was calculated from the difference in weights before processing and after cooling according to the formula:

(1) $$\mathrm{Wc}\;(\%)=\frac{\mathrm{MI}-\mathrm{MII}}{\mathrm{MI}}\;\times 100\%,$$

where Wc is the size of thermal drip (%), MI is the weight of the sample before thermal processing (g), and MII is the weight of the sample after cooling (g).

The forced drip of meat was determined using Grau and Hamm’s method [36] by placing a minced sample (about 300 mg) on Whatman paper No. 1. Both the paper and sample were placed between two glass plates and subjected to 5 kg of pressure for 5 min. On completion of the required squeezing time, the boundaries of the surface occupied by the sample of meat and the drip of meat juice were outlined on the paper and were subsequently planimeterized by using a digital planimeter. The measure of the size of forced drip of meat juice was the difference between both surfaces, which indicates water absorption capacity (cm^2^) of the meat sample (higher value corresponds to lower water absorption by the meat sample).

The shear force was measured using a TA.XT plus texturometer (Stable Micro Systems Ltd., Surrey, UK) equipped with a Warner–Bratzler shear blade with a triangular cut. The samples of raw meat were cut using a cylinder-shaped cork borer (with a diameter of 12.7 mm) along the muscle fibers. The samples prepared in this way were cut into sections, and the shear force (N/cm^2^) applied during the cutting process was recorded. Three technical repetitions were carried out per sample.

### 2.3. Statistical Analysis

All the experiments were performed in triplicate. The obtained results were assessed using statistical methods. Data were analyzed with the use of a three-way analysis of variance (ANOVA) in order to determine the differences in the selected physical and chemical properties of beef, which were found to be influenced by the slaughter method, gender, and type of muscle. For determining the effects of these parameters on the quality of the final product, the GLM (General Linear Model) procedure was used (ANOVA, STATIST ICA v. 13.1; StatSoft, Krakow, Poland) for a fixed-effect model with two types of slaughter, two groups of gender, and two groups of muscle. In the case of significant effects (*p* < 0.05), the average values were compared with Tukey’s post-hoc HSD test (ANOVA, STATISTICA v. 13.1; StatSoft, Krakow, Poland). Table 1, Table 2, Table 3 and Table 4 summarize the average values and standard error of the mean values of selected physical and chemical parameters of beef samples.

## 3. Results and Discussion

The type of slaughter technique and the muscle type showed a statistically significant effect on the pH_1_ value (Table 1). In addition, the type of slaughter method and gender, as well as the effect of interaction between these factors, demonstrated a statistically significant influence on features such as pH_24_ and pH_48_. Higher values of pH_1_ (*p* < 0.05) were observed in the meat samples of heifers and young bulls processed by the standard slaughter method. However, as early as 24 and 48 h after slaughter, higher pH values (*p* < 0.05) were observed in the meat sample of young bulls subjected to kosher treatment when compared to the raw sample obtained from carcasses of both genders prepared by the standard slaughter method. When analyzing the changes in the acidity of the meat of heifers, slightly higher values of pH_24_ and pH_48_ were observed in the raw material of the animals killed by kosher slaughter. However, these differences were found to be statistically insignificant. In the MS of cattle, lower pH_1_ values (*p* < 0.05) were noticed in comparison to the MLT of heifers and young bulls. The obtained range of pH values correspond to the range of values suggested for normal quality of meat. The pH_1_ value for RFN (red, firm, normal, and nonexudative) meat was found to be >6.3 (value above 5.8 is permissible), while the pH_24_ value was in the range of 5.5–5.7 (value up to 6.0 is permissible) [37].

Some authors [38,39] have shown that meat obtained from ritual slaughter is characterized by a high pH value after a longer storage period. D’Agata et al. [38] suggested that the pH values measured at 2 and 48 h after slaughter were similar (about 5.60) for the meat of cattle killed by Islamic ritual and conventional slaughter methods. In contrast, Holzer et al. [20] reported a lower pH for kosher meat compared to non-kosher meat. These authors, when analyzing the pH of the *longissimus lumborum* muscle of steers, concluded that the pH_24_ value of the meat subjected to the kosher process was 5.53, which was close to the average pH_24_ values obtained in their own studies on beef muscles (except for MLT and MS of kosher slaughter young bulls). Barrasso et al. [40], while examining the effect of religious slaughter on the pH and temperature of cattle carcasses, presume that higher pH_24_ values in animals subjected to ritual slaughter are associated with prolonged state of consciousness of the animal, which may be related to increased (i.e., longer-lasting) psychological stress and/or physical reactions, resulting in faster metabolism and increased glycogen consumption. Three hours after slaughter, the pH was at a comparable level (approx. 6.0) for traditional and kosher slaughter. The authors suggest that the animals slaughtered without stunning had a greater use of glycogen during bleeding, while pre-stunning the cattle reduced the risk of obtaining meat with a high ultimate pH. The stunned animals were probably less stressed and consumed less glycogen at the same time. Moreover, Niedźwiedź et al. [41] showed that the acidity value of *longissimus thoracis etlumborum* muscle in bull after 45 min of slaughter was 6.51, after 24 h (pH_24_) was 5.53, after 48 h (pH_48_) was 5.47, and after 72 h (pH_72_) was 5.47. The pH_48_ and pH_72_ values were lower in this study when compared to those obtained in their own research in the meat of cattle obtained from standard and kosher slaughter (Table 1). Moreover, the pH_24_ values were similar to the values obtained in our own research for the muscles of standard slaughter cattle and kosher slaughter heifers. Janiszewski et al. [42] showed that the pH_24_ values in bull’s *longissimus dorsi* muscle were in the range of 5.90–6.0. Pipek et al. [43] reported higher pH_24_ (6.02–6.08) values for the *longissimus lumborum et thoracis* muscle of heifers. Litwińczuk et al. [44] showed that the average pH_48_ value was 5.57.

Many authors [42,45] showed that the pH_48_ values ranged from 5.58 to 5.79, which was comparable to the pH in the meat of cattle from standard slaughter and some of the muscles of heifers from kosher slaughter. Other authors [43,46,47] showed higher pH_48_ values (5.71–5.99) in the meat of cattle. These values were similar to those obtained in the authors’ own research (Table 1) in the muscles of kosher bulls. According to Marenčić [25] and Węglarz [48], young bull’s meat in comparison to this raw material obtained from heifers carcasses is characterized, among others, by higher pH_u_ and pH_48_ values, respectively. Different results were reported by Miciński et al. [49], who found higher mean pH values than in their own studies in the *longissimus* muscle of Hereford and Limousine young bulls (6.25 and 6.58, respectively). The high pH values found in the present study indicated the presence of a DFD (Dark, Firm, Dry) defect in the tested raw meat [49]. Katsaras and Peetz [50], who studied morphological changes in dark cutting heated beef, found that fragmentation of myofibrils was greater in DFD meat and cooking losses were much smaller than in normal meat. These differences showed a greater tenderness of DFD meat compared to normal meat. However, it should be noted that DFD meat is characterized by a dark color at the muscle cut surface, and is drier compared to normal meat. Dark meat has limited durability because is more likely to be susceptible to microbial deterioration [47].

The interaction between the type of slaughter method and gender exhibited a statistically significant effect on the content of water of the beef sample (Table 2). Moreover, gender and muscle type influenced both the water and fat content of the carcass. On the other hand, gender and type of slaughter demonstrated a statistically significant influence on the protein content in the MLT and MS of cattle. Higher fat content and lower water content were noticed (*p* < 0.05) in both the MLT and MS of heifers, in comparison to the concentration of these compounds in the same muscles obtained from young bull carcasses regardless of the type of slaughter. However, the muscles of young bulls had a higher content of protein (*p* < 0.05) than the muscle tissue obtained from carcasses of heifers, regardless of slaughter type. Higher protein content was observed (*p* < 0.05) in both the MLT and MS of cattle killed by standard slaughter, when compared to the raw beef sample killed by ritual slaughter. This could be, for example, due to the greater amount of blood remaining in blood vessels of the muscles of standard slaughter cattle.

Considering the chemical composition of beef, higher water content and lower fat content were demonstrated in the MS of young bulls and heifers than in the MLT of cattle regardless of slaughter type. In the meat of kosher heifers, the water content was statistically significantly (*p* < 0.05) correlated with the fat content (r = −0.99) and protein content (r = 0.83).

Sakowski et al. [51] found the protein content (20.1%) in the *longissimus dorsi* muscle of Hereford young bulls to be similar to the average protein content obtained in the present study in both the MLT and MS of young bull carcasses obtained from standard slaughter. These authors reported a higher water content (73.5%) and lower fat content (5.2%) than the amounts obtained in their own research studies in the MLT of cattle killed by both types of slaughter methods and in the MS of heifers from kosher slaughter. In the same muscles of young bulls classified as post-slaughter class R, Wajda et al. [52] showed a higher average protein (23.67%) and lower fat content (1.35%) than that determined in the beef muscles in their own research. Choroszy et al. [53] reported higher protein (22.43%) and lower fat content (1.48%) in the MLT of Limousine bulls than the amounts obtained in the beef muscles in their own research. Nowak et al. [54] suggested higher protein content (20.67%) in the biceps femoris muscle of heifers than that obtained in their own research in the muscles of cattle obtained from both types of slaughter methods. Moreover, the authors demonstrated lower fat content (2.23%) and higher water content (75.38%) than that observed in their own research study in the muscles of cattle from both types of slaughter. Florek et al. [55] showed that the protein content in *longissimus lumborum* muscle of young bulls (21.94%) and heifers (21.12%) was higher than in the beef sample in their own research. Moreover, these authors found a higher water content (74.81%) in the bull muscle than in their own research studies in MLT obtained from the carcasses of same-gender animals killed by both types of slaughter methods. Moreover, the water content in the muscle of heifers was higher (73.91%) than that obtained in the authors’ own research on the muscles of heifers, except for the MS of the same-gender animals killed by standard slaughter. The fat content in the meat of young bulls (1.07%) and heifers (2.85%) was lower than that obtained in their own study on the muscles of animals of the same gender.

Gender has a significant influence on the quality characteristics of the carcass. The influence of sex (female, male, castrated) of ruminants is mainly related to the amount of fat deposited and its location, growth rate, and carcass efficiency [56]. Testosterone—an androgenic hormone produced by male testicular interstitial cells—has a positive effect on muscle development in this sex. Pre-pubertal castration interrupts the androgen formation process and the animal’s growth rate is delayed. On the other hand, heifers, compared to young bulls, get fat earlier and more often, have less developed valuable body parts, and are less muscular. Their meat, however, shows better marbling, fine-grained muscle structure, lower shear force, and thus is juicier, tender, and aromatic. Female hormones cause slower formation of connective tissue, which has a positive effect on the tenderness of the meat of heifers. The lower tenderness of meat of young bulls is caused by both a higher share of collagen and an increased level of calpastatin (protease inhibitor inhibiting the post-mortem tenderizing process) [8].

The type of slaughter method and muscle type were the major factors that influenced the composition of minerals (Table 3). In addition, gender, type of slaughter, and the effect of interaction between these two factors had a statistically significant influence on the salt content. A higher content of minerals and salt (*p* < 0.05) was observed in the muscles of cattle obtained through kosher slaughter than in the raw material obtained from carcasses of animals subjected to standard slaughter. Moreover, in the MS of heifers from ritual slaughter, the content of minerals (*p* < 0.05) was found to be twice the amount determined in the raw material obtained from animals of the same gender from standard slaughter. Furthermore, higher mineral content was observed in the MLT of cattle than that obtained for the MS (except for heifers from ritual slaughter) (*p* < 0.05). The salt concentration in the muscles of young bulls from kosher slaughter was almost double (*p* < 0.05) in comparison to the carcasses of animals of the same gender killed by the standard slaughter method. This finding is most likely due to a 10-fold increase in the sodium content after processing of the meat by koshering method, which was found to be consistent with other studies.

Domaradzki et al. [57] showed ash content of 1.03% and 1.05%, respectively, in the *longissimus lumborum* and *semitendinosus* muscles of young slaughter cattle. Śmiecińska and Wajda [58] reported that the ash content in the *longissimus dorsi* muscle of cows was in the range of 1.19–1.28%, and Florek et al. [55] showed the values to be 1.24% and 1.22% in the *longissimus lumborum* muscle of young bulls and heifers, respectively. The values obtained by these authors were higher than in their own research studies in the muscles of cattle, with the exception of the MLT of young bulls and the MS of heifers from kosher slaughter. Zając et al. [59] found that the content of total collagen in raw beef meat of heifers ranged from 0.29% to 0.96%. The values obtained by the cited authors were lower than the amounts observed in the beef muscles in their own research. Domaradzki et al. [60] showed that the mean values of total collagen in the *longissimus* muscle of the lumbar spine of heifers and young bulls were 8.91 and 10.55 mg/g, respectively, and in the *semitendinous* muscle were 12.13 and 16.58 mg/g, respectively. Chriki et al. [61] indicated that the total average collagen content in the MLT of young bulls and cows was 3.3 and 2.8 mg/g dry weight, respectively. However, the insoluble collagen fractions were found to be 2.9 and 2.3 mg/g dry weight, respectively.

Collagen is the main component of intramuscular connective tissue, and its composition and content are responsible for the hardness of cooked meat [62]. The researchers found that the collagen content was higher in dairy cattle and early-maturing meat breeds compared to late-maturing breeds [63,64]. The type of slaughter method and the effect of interaction between the type of slaughter and gender had a statistically significant effect on forced drip, thermal drip, and shear force values of the beef sample (Table 4). Moreover, gender and muscle type affected the variations in shear force. Statistically significant differences in the shear force values were observed between the MS and MLT of cattle obtained by standard slaughter and that of young bulls obtained by kosher slaughter. Higher values of the shear force (*p* < 0.05) were demonstrated in the MS of cattle than in the MLT of animals. Higher values of shear force (*p* < 0.05) were demonstrated in both MLT and MS of young bulls compared to the meat of heifers, regardless of the type of slaughter. Moreover, higher values of the shear force (*p* < 0.05) were found in the muscles of animals from standard slaughter in comparison with the values obtained in the muscles of cattle from kosher slaughter.

The tenderness of meat is influenced by the physical and chemical properties of muscles. Many factors of the muscle structure itself significantly affect the texture characteristics of meat, the most important of which are the amount and degree of cross-linking of connective tissue, the length of the sarcomeres, the speed and degree of post-mortem proteolysis, and the content and proportions of the types of muscle proteins—myofibrils and sarcoplasmic proteins. Individual muscles within one carcass differ in terms of tenderness, which results from the intensity of their work performed during life, affecting the thickness of the fibers and the collagen content. Muscles that did not perform substantial work during the life of animals are characterized by a lower content of collagen. Very active muscles contain relatively large amounts of this protein [65,66]. The tenderness of the meat is greater when the cross-section of the muscle fibers is smaller, and the smaller the bundles of these fibers, the greater the length of the sarcomeres. The shortening of sarcomeres may be a consequence of improperly carried out meat conditioning and maturation process [67]. Heifers, compared to young bulls shows better marbling, fine-grained muscle structure, lower shear force and thus, their meat is juicier and more tender [8].

Agbeniga et al. [68] showed statistically significant higher values of cooking losses in cattle from conventional slaughter compared to the muscles of animals obtained from kosher slaughter. Moreover, the authors found no effect of the slaughter method on meat drip (drip loss). Vergara and Gallego [69] also showed no statistically significant differences in the case of driploss between unstunned and electrically stunned lambs.

pH plays a key role in shaping meat drip [68]. Agbeniga et al. [68] found that a similar pH profile of meat obtained from cattle carcasses from kosher and standard slaughter may be the reason for the lack of statistically significant differences in driploss. In addition, stress during stunning can cause physiological changes, including redistribution of visceral blood towards the brain and skeletal muscles, thereby causing greater cooking loss [70].

Nowak et al. [54] found the value of the shear force in the biceps femoris muscle of heifers to be 41.8 N/cm^2^. The lowest values of the shear force were found in the infraspinatus muscle (32.1 N/cm^2^) and the highest in the *semimembranosus* muscle (49.1 N/cm^2^). Śmiecińska et al. [71] reported the shear force value of 33.68 N/cm^2^ in the *longissimus lumborum* muscle of young bulls. Domaradzki et al. [72] calculated the mean values of the shear force to be 117.9 N/cm^2^ in the *longissimus lumborum* muscle of Polish Red bulls, while the value was 125.6 N/cm^2^ in the meat of the white-backed young bulls. Niedźwiedź et al. [41] determined the values of the shear force 48 h after slaughter in the bull’s *longissimus thoracis et lumborum* muscles to be 81.6 N/cm^2^. Similar values of shear force (78.65 N/cm^2^) were reported by Niedźwiedź et al. [73] in the same muscles of young slaughter cattle. On the other hand, Bureš and Bartoň [64] showed that the average value of force was 58.6 N/cm^2^ in *the longissimus lumborum* muscle of Holstein bulls, which was similar to that obtained in our research in the *longissimus thoracic* muscle of young bulls from standard slaughter. However, the values of this parameter in the meat of Fleckvieh bulls (49.8 N/cm^2^) were slightly higher than those observed in the authors’ own research in the MLT of heifers from standard slaughter and young bulls from kosher slaughter. Rudy et al. [74] obtained a lower shear force value (57.11 N/cm^2^) in the longest back muscle of young bulls compared with the authors’ own research in the meat of same-gender animals killed by standard slaughter. On the other hand, the authors determined the value to be 48.53 N/cm^2^ in the muscle of heifers, which is lower than in the MLT of heifers and higher than in the MS of animals of the same gender from both types of slaughter.

Agbeniga et al. [68] report that there are many reasons responsible for the difference in shear force between meat samples obtained from the two methods of slaughter. First, the amount of water bound in the fibers of the meat can affect tenderness. Higher values of cooking loss of meat from conventional slaughter animals may contribute to higher values of shear force. In addition, carcass temperature 24 h after slaughter and a faster rate of its decrease in cattle from conventional slaughter may play a role in sarcomeres shortening and cause cold shortening, which may therefore lead to higher shear force values in the muscles of animals obtained from this type of slaughter.

When the hydration properties of beef were analyzed, young bulls and heifers from kosher slaughter presented lower values (*p* < 0.05) than the raw material obtained from cattle carcasses from standard slaughter. More favorable hydration properties of kosher beef can be attributed to the higher pH_48_ values. The pH has a very strong influence on the water-holding capacity and tenderness of meat [75]. In other studies, which are not yet published, no statistically significant correlation coefficients were found between pH and water content, protein content, and hydration properties of the MLT muscle obtained from carcasses of young bulls from standard slaughter. On the other hand, in the same muscle obtained from young bull carcasses from kosher slaughter, statistically significant (*p* < 0.05) correlation coefficients were found between pH_24_ and forced drip (r = −0.91) and pH_48_ and thermal drip (r = −0.83).

Rudy et al. [74] determined the mean value of forced drip during refrigerated storage after 48 h of slaughter in the longest back muscle of heifers, which was found to be 6.10%, but in the case of bull meat, the value was 4.40%. Wajda et al. [52] determined the average water absorbability values to be 5.77 cm^2^. These values were higher than those obtained in the meat of cattle from kosher slaughter, but lower than those found in the muscles of cattle from standard slaughter. Chávez et al. [76] obtained higher values of thermal drip in the meat of cattle Bos taurus and Bos indicus, that is, 34.32% and 36.27%, respectively, than in their own research. Similar results were obtained by Niedźwiedź et al. [53], who determined that the value of thermal drip in *longissimus thoracis et lumborum* muscles after 48 h of slaughter was 33.13%. On the other hand, Rudy et al. [74] determined the mean values of thermal drip in the muscles of heifers after 48 h of slaughter to be similar (23.90%) to the results obtained in their own research in the MLT of same-gender animals from ritual slaughter. Moreover, in the muscles of young bulls, these authors obtained lower values for this parameter (22.80%) than in beef in their own research. Domaradzki et al. [72] showed similar results for this parameter in the *longissimus lumborum* muscle of young Polish Red bulls (28.82%).

## 4. Conclusions

The results indicate that all the investigated factors play a role in differentiating the quality of beef. Lower early post-mortem pH values and higher pH values after 24 and 48 h following slaughter were observed in the meat of kosher young bulls, which was most likely caused by the prolonged state of consciousness of the animal (in ritual slaughter), which may be related to increased (i.e., longer-lasting) psychological stress and/or physical reactions, resulting in faster metabolism and increased glycogen consumption during bleeding. Kosher slaughter, and the closely related koshering process, resulted in a decrease in the forced and thermal drip values of beef, but the values did not show gender-based differences. More favorable water-holding capacity related properties of kosher beef can be attributed to the higher pH values, which may result in lower microbiological stability of such raw material. Considering the chemical composition of beef, higher fat and lower water contents were obtained in the muscles of heifers compared to young bull. This may be because heifers, compared to young bulls, likely become fatter and stronger earlier on, and female hormones also cause a slower formation of connective tissue, which has a positive effect on the tenderness of their meat, resulting in better marbling, fine-grained muscle structure, and a lower cutting force in the meat of heifers. Moreover, higher fat and lower water content was obtained in the longest thoracic muscle in comparison to the amounts of these components determined in the supraspinatus muscle. It was also found that the shear force was higher in MS, which could be due to the lower fat content and slightly higher collagen content in this muscle. Muscles that do not perform much work during the life of the animals (e.g., MLT) are usually characterized by a lower collagen content and a fine fiber structure.

## Figures and Tables

**Table 1:** Changes in the pH values of beef depending on the type of slaughter, muscle type, and gender of cattle.

Specification	Muscle Type	Standard Slaughter		Kosher Slaughter		SEM	ANOVA			
		Young Bulls $\bar{x}$	Heifers $\bar{x}$	Young Bulls $\bar{x}$	Heifers $\bar{x}$		S	M	G	S × G
pH_1_	MLT	6.99 ^a^	6.98 ^a^	6.42 ^Ab^	6.73 ^A^	0.268	*	*		
	MS	6.98	6.96	6.31 ^B^	6.65 ^B^	0.315	*	*		
pH_24_	MLT	5.58 ^a^	5.55 ^a^	5.83 ^b^	5.57 ^a^	0.132	*		*	*
	MS	5.51	5.50	5.81	5.61	0.144	*		*	*
pH_48_	MLT	5.68 ^a^	5.64 ^a^	5.99 ^b^	5.77 ^a^	0.156	*		*	*
	MS	5.61	5.60	5.95	5.73	0.163	*		*	*
pH_72_	MLT	5.70	5.71	5.80	5.61	0.078				
	MS	5.68	5.69	5.75	5.62	0.053				

Notes: ^a,b^ Differences marked in the rows with statistically significant values at the level *p* < 0.05 according to Tukey’s HSD test. ^A,B^ Differences marked in the columns only between the muscles with statistically significant values at the level *p* < 0.05 according to Tukey’s HSD test. No letters or the same letters mean no statistically significant differences. ANOVA: three-factor analysis of variance between the type of slaughter (S), gender (G), and muscle (M). * *p* < 0.05. MLT: *longissimus thoracis* muscle; MS: *supraspinatus* muscle.

**Table 2:** Basic chemical composition (% fresh muscle tissue) of beef depending on the type of slaughter, muscle type, and gender of cattle.

Specification	Muscle Type	Standard Slaughter		Kosher Slaughter		SEM	ANOVA			
		Young Bulls $\bar{x}$	Heifers $\bar{x}$	Young Bulls $\bar{x}$	Heifers $\bar{x}$		S	M	G	S × G
Water (%)	MLT	70.37 ^a^	67.01 ^Aa^	70.82 ^a^	61.81 ^Ab^	4.158		*	*	*
	MS	74.93 ^a^	74.48 ^Ba^	74.63 ^a^	69.27 ^Bb^	2.711		*	*	*
Protein (%)	MLT	20.48 ^a^	19.20 ^a^	19.65 ^a^	17.79 ^b^	1.126	*		*	
	MS	20.00 ^a^	19.60	19.17 ^b^	18.92 ^b^	0.477	*		*	
Fat (%)	MLT	7.46 ^Aa^	11.77 ^A^	7.29 ^Aa^	17.71 ^Ab^	4.896		*	*	
	MS	2.75 ^Ba^	3.60 ^B^	2.92 ^Ba^	8.35 ^Bb^	2.656		*	*	

Notes: ^a,b^ Differences marked in the rows with statistically significant values at the level *p* < 0.05 according to Tukey’s HSD test. ^A,B^ Differences marked in the columns only between the muscles with statistically significant values at the level *p* < 0.05 according to Tukey’s HSD test. No letters or the same letters mean no statistically significant differences. ANOVA: three-factor analysis of variance between the type of slaughter (S), gender (G), and muscle (M) * *p* < 0.05. MLT: *longissimus thoracis* muscle; MS: *supraspinatus* muscle.

**Table 3:** Content of minerals, collagen, and salt (% fresh muscle tissue) in beef depending on the type of slaughter, muscle type, and gender of cattle.

Specification	Muscle Type	Standard Slaughter		Kosher Slaughter		SEM	ANOVA			
		Young Bulls $\bar{x}$	Heifers $\bar{x}$	Young Bulls $\bar{x}$	Heifers $\bar{x}$		S	M	G	S × G
Minerals (%)	MLT	0.98 ^A^	0.90 ^a^	1.49 ^Ab^	1.15	0.262	*	*		
	MS	0.81 ^B^	0.85 ^a^	0.99 ^B^	1.45 ^b^	0.294	*	*		
Collagen (%)	MLT	1.92	1.82	2.05	1.90	0.095				
	MS	2.06	2.05	1.97	1.95	0.056				
Salt (%)	MLT	0.25 ^a^	0.40 ^b^	0.49 ^b^	0.46 ^b^	0.107	*		*	*
	MS	0.35 ^a^	0.56 ^b^	0.61 ^b^	0.59 ^b^	0.120	*		*	*

Notes: ^a,b^ Differences marked in the rows with statistically significant values at the level *p* < 0.05 according to Tukey’s HSD test. ^A,B^ Differences marked in the columns only between the muscles with statistically significant values at the level *p* < 0.05 according to Tukey’s HSD test. No letters or the same letters mean no statistically significant differences. ANOVA: three-factor analysis of variance between the type of slaughter (S), gender (G), and muscle (M) * *p* < 0.05. MLT: longissimus thoracis muscle; MS: supraspinatus muscle.

**Table 4:** Water-holding capacity related properties and shear force of beef depending on the type of slaughter, muscle type, and gender of cattle.

Specification	Muscle Type	Standard Slaughter		Kosher Slaughter		SEM	ANOVA			
		Young Bulls $\bar{x}$	Heifers $\bar{x}$	Young Bulls $\bar{x}$	Heifers $\bar{x}$		S	M	G	S × G
Shear force (N/cm^2^)	MLT	59.92 ^Aa^	48.05 ^Ab^	48.54 ^Ab^	44.62 ^b^	6.657	*	*	*	*
	MS	74.92 ^Ba^	62.86 ^B^	64.23 ^Bb^	49.84 ^b^	10.276	*	*	*	*
Forced drip (cm^2^)	MLT	7.21 ^a^	6.50 ^ac^	4.98 ^b^	5.54 ^bc^	0.992	*			*
	MS	7.96 ^a^	7.81 ^a^	5.05 ^b^	4.67 ^b^	1.754	*			*
Thermal drip (%)	MLT	28.93 ^a^	25.11 ^b^	23.58 ^b^	23.81 ^b^	2.475	*			*
	MS	31.56 ^a^	27.27 ^b^	24.51 ^b^	25.33 ^b^	3.149	*			*

Notes: ^a,b,c^ Differences marked in the rows with statistically significant values at the level *p* < 0.05 according to Tukey’s HSD test. ^A,B^ Differences marked in the columns only between the muscles with statistically significant values at the level *p* < 0.05 according to Tukey’s HSD test. No letters or the same letters mean no statistically significant differences. ANOVA: three-factor analysis of variance between the type of slaughter (S), gender (G), and muscle (M). * *p* < 0.05. MLT: *longissimus thoracis* muscle; MS: *supraspinatus* muscle.

## Data Availability

The authors declare that data or models are not deposited in an official repository.
